# A Possible Mechanism for Double-Yolked Eggs in the Early Stage of Egg-Laying in Zhedong White Goose–Function of IGF1 and LHR Signaling

**DOI:** 10.3390/ani12212964

**Published:** 2022-10-28

**Authors:** Jie Liu, Xingfei Zhao, Zichun Dai, Pengxia Yang, Rong Chen, Binbin Guo, Mingming Lei, Zhendan Shi

**Affiliations:** 1 Institute of Animal Science, Jiangsu Academy of Agricultural Sciences, Nanjing 210014, China; 2 Key Laboratory of Crop and Livestock Integration, Ministry of Agriculture, Nanjing 210014, China

**Keywords:** goose, IGF1, LHR, double-yolked egg, first egg-laying cycle

## Abstract

**Simple Summary:**

The reason that birds produce double-yolked eggs is not clear. However, double-they often occur in the early egg-laying stage. We detected and recorded the proportion of double-yolked eggs, the number of abdominal follicles, and the changes of key ovulation-related genes in Zhedong white geese. We proposed that, in the first egg-laying stage of geese, high plasma concentrations of insulin like growth factor 1 (IGF1) stimulate the development of pre-hierarchal follicles, causing more than one follicle to be selected at the same time, to mature at the same rate under the same gonadotrophin milieu, and to ovulate at the same time to produce double-yolked eggs.

**Abstract:**

The cause of double-yolk (DY) egg production in birds is unclear, but it is related to body weight and adiposity. We explored the causes of the high proportion (up to 26%) of DY eggs in the first clutch of Zhedong white geese. We recorded the egg production of Zhedong white geese during the first egg-laying cycle and counted the proportion of DY eggs. We found that 30% of geese had 3 sets of double or triple follicles of the same diameter in the abdomen, which was close to the DY egg rate. In addition, the mRNA expression levels of the steroidogenic acute regulatory protein (*StAR*) and luteinizing hormone receptor (*LHR*) genes in granulosa cells were similar within the same set of follicles. Furthermore, the IGF1 concentration in geese that had at least 3 sets of follicles of the same diameter was significantly higher than that in birds with 0–1 set of follicles of the same diameter. Thus, we proposed that, in the first egg-laying stage of geese, high plasma concentrations of IGF1 stimulate the development of pre-hierarchal follicles and cause more than one follicle to be selected at the same time, mature at the same rate under the same gonadotrophin milieu, and ovulate at the same time to produce DY eggs.

## 1. Introduction

In the process of laying eggs, birds generate a certain proportion of abnormal eggs, such as soft-shell eggs, double-yolked (DY) eggs, and non-yolked eggs [1]. DY eggs have higher nutritional and commercial value than ordinary eggs because of the high proportion of egg yolks and good meaning, culturally [2]. Current speculation posits that the causes of DY eggs include physiological, genetic, and pathological factors [3,4,5]. Production experience suggests that the proportion of DY eggs produced by primary poultry is high; however, no research has been undertaken to elucidate the molecular mechanism.

The follicular sequence of birds has a decisive effect on egg-laying activities. Once follicular development completely loses order, it leads to adverse events, such as a decline in egg laying rate or even production suspension. For example, broiler breeders are not feed-restricted, which results in excessive weight gain, causing the follicles in the abdominal cavity to be disordered; despite a high number of follicles, these birds cannot lay eggs [6]. There is a state between order and disorder that occurs in primiparous females. At this time, the female is overnourished, and a high level of reproductive hormones leads to a high proportion of DY egg production. In addition, by observing the status of follicles in the abdominal cavity, it was found that there were one or more pairs of follicles with the same diameter. The yolk diameters of DY eggs are consistent [7], so it is speculated that follicles with similar diameters in the abdominal cavity may form DY eggs in subsequent egg-laying.

In the early stage of the first egg-laying, we found that the proportion of DY eggs produced in the Zhedong white geese breeding season was as high as 26% and that there were one or more pairs of follicles with similar diameters in the abdominal cavity. It has been speculated that follicles with similar diameters eventually produce DY eggs, but it is not clear why there are a large number of follicles with similar diameters at this stage and why follicles with similar diameters can ovulate at the same time to form DY eggs. Therefore, this study analyzed the follicular function during the first egg-laying cycle of Zhedong white geese during the breeding season and revealed the possible mechanism of DY egg production.

## 2. Materials and Methods

### 2.1. Ethical Approval

The experimental procedures were approved by the Research Committee of Jiangsu Academy of Agricultural Sciences and conducted with adherence to the Regulations for the Administration of Affairs Concerning Experimental Animals (Decree No. 63 of the Jiangsu Academy of Agricultural Science on 8 July 2014).

### 2.2. Animals and Experimental Procedure

**Figure 1:**
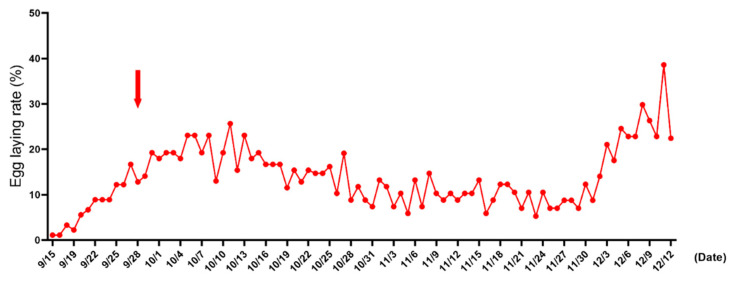
Egg laying rate of Zhedong white geese. The red arrow represents the sampling time point.

The experiment was conducted in June 2021. Zhedong white geese of the same batch and source, aged 200 days, were selected as the research subjects. The male:female ratio was 1:4. All experimental geese were randomly divided into four groups with a feeding area of 1 m^2^. During this period, the geese drank freely and were mechanically ventilated longitudinally. In the early stages of the experiment, the geese were exposed to natural light, and eggs were found sporadically. To ensure the accuracy of the experiment, light control technology was used to regulate breeding geese to resume production. First, in late June, the breeding geese quickly stopped laying eggs when a long light program (light:dark = 19 h:5 h) combined with feeding restriction measures (100 g per goose per day; reserve goose feed, 802; COFCO, Beijing, China) were utilized. After continuous treatment, the ovaries of the breeding geese degenerated to the size of ovaries in the non-breeding season. The short light program (light:dark = 7 h:17 h) and the second long light program (light:dark = 11 h:13 h) was used in combination with the measures of free feeding (reserve goose feed, 803; early egg laying period goose feed, 806; COFCO, Beijing, China) during the egg-laying period to allow the ovaries of breeding geese to enter the recovery stage. After approximately 20 days of treatment, production resumed in mid-September. On 28 September, 10 female geese with eggs were randomly selected (Figure 1). The number of follicles was recorded, and the longest diameter of the follicles was measured using a Vernier caliper. Blood and granulosa cells of the follicles were collected and quickly stored in liquid nitrogen.

### 2.3. RNA Isolation, cDNA Synthesis, and Real-Time PCR

Total RNA was isolated from granulosa cells using 0.5 mL TRIzol reagent (Vazyme, Nanjing, China). One microgram of total RNA was treated with RNase-free DNase and reverse-transcribed into cDNA using random hexamer primers (Vazyme, Nanjing, China). One microliter of diluted cDNA (1:10, *v*/*v*) was used for real-time PCR. Technical variations were normalized using γ-DH as an internal control. Primers for real-time PCR (Table 1) were synthesized by Tsingke Biotech (Nanjing, China). The 2^−^^△△CT^ method was used to analyze the results, and gene mRNA levels were expressed as the fold-change relative to the mean value of the first group.

### 2.4. IGF1 Concentration Measurements

IGF1 levels in the plasma and liver were measured using a commercial IGF1 assay kit (goose-IGF1) purchased from MLBio, Nanjing, China, according to the manufacturer’s instructions.

### 2.5. Statistical Analysis

The results, presented in Figures 5 and 6 as means ± SEM, were analyzed using independent samples *t*-test and one-way ANOVA with SPSS (SPSS 22.0, SPSS Inc., Chicago, IL, USA). Statistical significance was set at *p* < 0.05.

## 3. Results

### 3.1. Laying Rate during the First Clutch of Egg-Laying

The geese began to lay eggs on September 15 and reached the peak of laying around October 10, with a laying rate of 27%. Then, the laying rate decreased, and some geese gradually developed incubation behavior before entering the second clutch of egg-laying at the end of November (Figure 1).

### 3.2. Ovarian Appearance and Diameter

**Figure 2:**
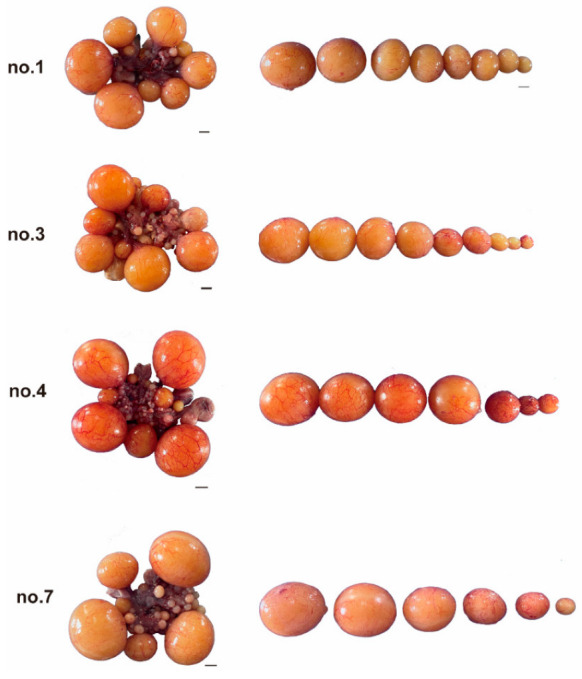
Morphology of ovary and follicle during the first clutch of egg-laying. Scale = 1 cm.

**Figure 3:**
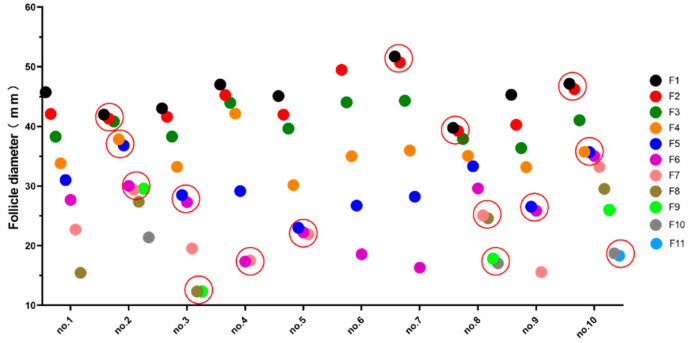
Follicular diameter in the first egg-laying cycle of the Zhedong white goose. The follicles enclosed within the circles are supposed to develop to produce double-yolked eggs.

There were 6–11 large yellow follicles in the abdominal cavity of geese at the early stage of the first laying period, and one or more pairs of follicles with the same diameter (diameter difference < 1.20 mm) were found in some geese (Figure 2 and Figure 3). The largest follicle diameter was measured. The diameter of F1 ranged from 39.75 mm to 51.72 mm, and the diameter of the smallest large yellow follicles ranged from 12.33 mm to 21.89 mm.

### 3.3. Relationship between Expression Levels of LHR and StAR and Follicle Diameter

**Figure 4:**
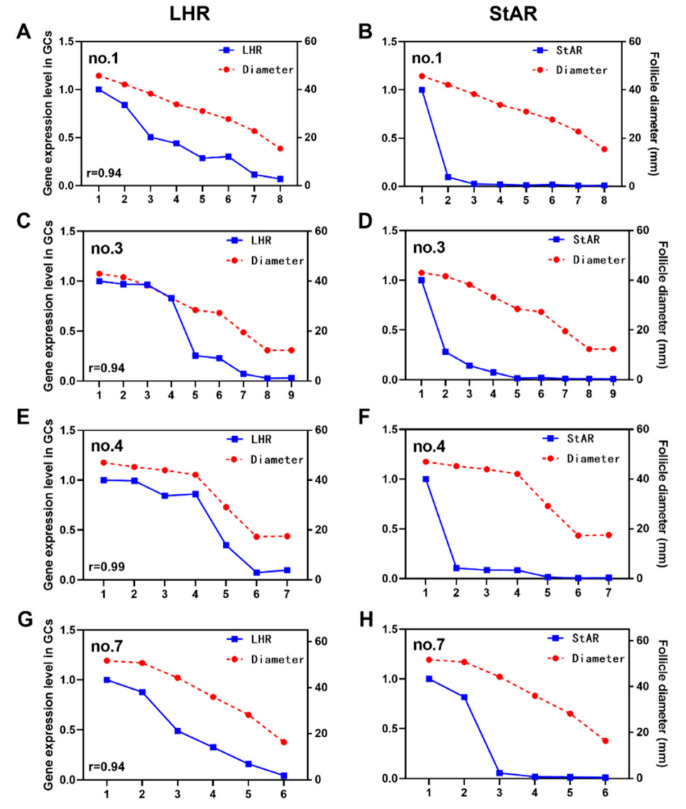
*LHR* (**A**,**C**,**E**,**G**) and *StAR* (**B**,**D**,**F**,**H**) expression and follicle diameter in granulosa cells (GCs).

The expression level of LHR decreased with the decrease in follicle diameter, and the greater the follicle diameter, the higher the expression of LHR. This suggested that the LH signal intensity received by the two follicles with similar diameters was consistent. In addition, the expression of StAR had an obvious pattern, and the expression level in F1 was significantly higher than that in the other follicles. However, it is worth noting that no.7 F1 and F2 were very similar in diameter, as were the expression levels of StAR (Figure 4H).

### 3.4. IGF1-Related Gene Expression and IGF1 Concentration in the Blood

**Figure 5:**
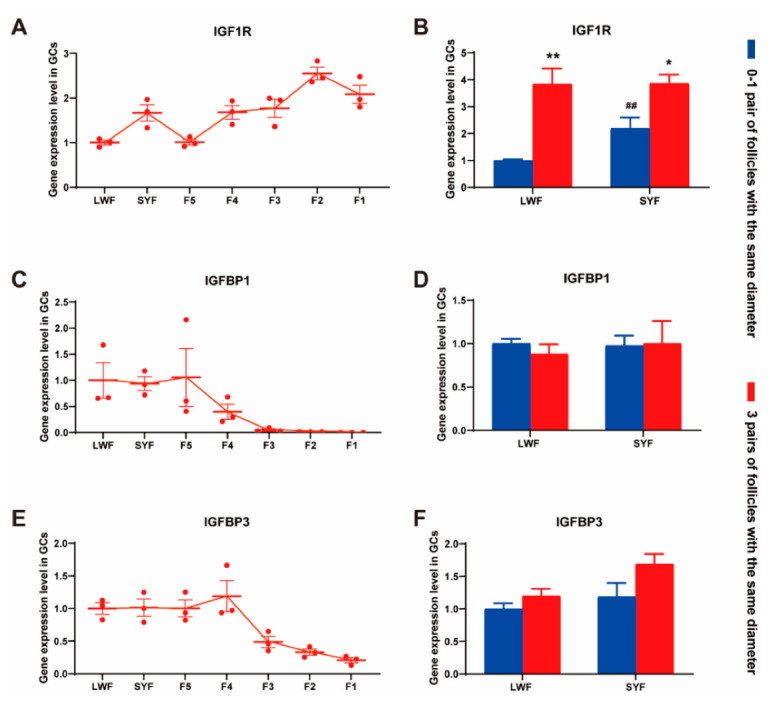
IGF1-related gene expression in granulosa cells. (**A**,**C**,**E**) *IGF1R*, *IGFBP1* and *IGFBP3* expression in granulosa cells of different follicles. Data are expressed as mean ± SEM (n = 3). (**B**,**D**,**F**) *IGF1R*, *IGFBP1* and *IGFBP3* expression in granulosa cells of small follicles divided into two groups (0–1 pair group and 3 pairs group). Data are expressed as mean ± SEM. * *p* < 0.05 and ** *p* < 0.01, compared to 0–1 pair group; ## *p* < 0.01, compared to LWF group.

The results of RNA-seq (data from other experiments using Yangzhou geese) showed that the expression of *IGF1R* mRNA increased with the development and maturation of follicles. *IGFBP1/3* was expressed at high levels in small follicles (large white follicles (LWFs) and small yellow follicles (SYFs)), but its expression was significantly decreased in F1-F3 follicles (Figure 5A,C,E).

According to the number of follicle pairs with the same diameter, Zhedong white geese were divided into the 0–1 pair group and 3 pairs group. The 0–1 pair group included no. 1/4/5/6/7/9 geese, and the 3 pairs group included no. 2/8/10 geese. The mRNA abundance of *IGF1*, *IGFBP1*, and *IGFBP3* in LWF and SYF granulosa cells was detected. The results showed that the expression of *IGF1R* in SYF granulosa cells of the 0–1 group was higher than that in the LWF group. In addition, whether LWF or SYF, the expression of *IGF1R* in the 3 pairs group was significantly higher than that in the 0–1 pair of groups (Figure 5B). There were no differences in *IGFBP1* and *IGFBP3* levels (Figure 5D,F).

**Figure 6:**
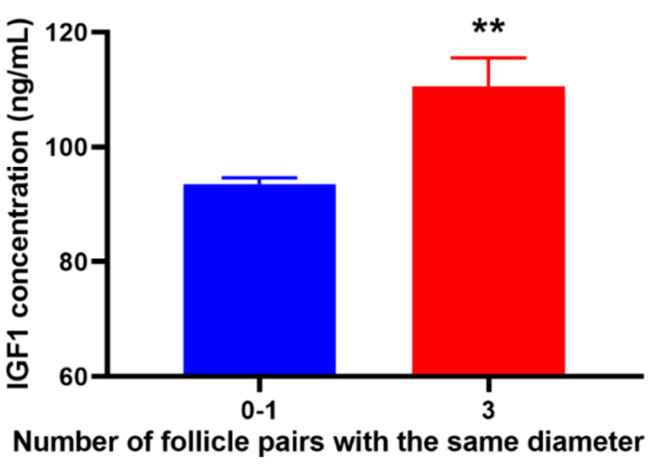
Plasma concentration of IGF1 in geese with different numbers of paired follicles. Data are expressed as mean ± SEM. ** *p* < 0.01.

We also found that geese with three pairs of follicles of the same diameter had a higher IGF1 content in the serum (Figure 6).

## 4. Discussion

The follicular sequence of birds has a decisive effect on egg-laying activities. When follicular development completely loses order, it leads to adverse events, such as a decline in the egg laying rate or even production suspension [8,9,10]. For example, broiler breeders are not food-restricted, which results in excessive weight gain; therefore, the follicles in their abdominal cavities are in a disordered state. Although broiler breeders have a large number of follicles, they still have a low egg-laying rate [6]. Primiparous females are in a state between order and disorder. At this stage, the female is overnourished, and a high level of reproductive hormones leads to a high proportion of DY eggs. In addition, by observing the status of follicles in the abdominal cavity, it was found that there were one or more pairs of follicles with the same diameter. The basic yolk diameter of DY eggs are the same [11]. Therefore, it has been speculated that follicles with similar diameters in the abdominal cavity may form DY eggs in subsequent egg-laying activities.

The occurrence of DY eggs is common in birds. DY eggs are formed when two yolks ovulate within three hours and become enclosed in one egg [12,13]. In addition, DY eggs are more prevalent at the onset of laying [7]. At present, research on DY eggs mainly focuses on the non-destructive identification of DY eggs [2,12] and their fertilization [14]. However, the reason for the high rate of DY eggs at laying onset has remained unclear.

In this work, we found that the rate of DY eggs in the first egg-laying cycle of the Zhedong white goose breeding season was 26% (15–30 September, a total of 115 eggs), and that there were one or more pairs of follicles with similar diameters in the abdominal cavity. In addition, the expression of LHR in granulosa cells of all levels of follicles in the abdominal cavity not only followed the law of gradual increase, but was also highly correlated with follicle diameter (average correlation coefficient: 0.94, n = 10). Therefore, follicles with similar diameters received similar LH stimulation signals. 

During ovulation, the follicular zone is likely to rupture simultaneously. In such cases, two eggs are swallowed by the infundibulum and go through various parts of the reproductive tract, eventually forming DY eggs. Interestingly, the high expression of *StAR* in F1 before ovulation showed three different forms: the diameters of F1 and F2 were quite different, while the expression of *StAR* in F1 was more than 10 times that of F2 (e.g., no.1). The diameters of F1 and F2 were similar, and the expression of *StAR* in F1 was 5–10 times that in F2 (e.g., no.3/4). The diameters of F1 and F2 were very similar (diameter difference < 1.20 mm), and the expression levels of *StAR* in F1 and F2 were also similar (e.g., no.7). The expression of StAR is mainly regulated by the LH cAMP signal [15]; therefore, the expression level of StAR largely reflects the response of follicles to LH. The LH peak before ovulation was the main stimulation for F1 follicular germinal vesicle rupture and subsequent ovulation [16,17]. Poultry usually produces a circulating LH peak 4–6 h before ovulation, and the receptor (LHR) gradually increases with the maturation of follicles, reaching the highest level in F1 follicles [18,19]. During the LH peak, F1 follicles express high levels of StAR and secrete large amounts of progesterone [20,21]. Therefore, the expression of LHR and StAR in preovulatory follicles can directly reflect the sequence of ovulation. Our results supported the idea that follicles with similar diameters receive more consistent LH signals and have a higher possibility of simultaneous rupture of the follicular zone when the LH peak occurs before ovulation, at which time DY eggs are produced.

In this study, we also found that geese with three pairs of follicles of the same diameter had a higher IGF1 content in the serum, and the expression level of *IGF1R* in granulosa cells was higher than that in geese with no pairs or only one pair of follicles with the same diameter. IGF1 is mainly synthesized in the liver and influences whole-body metabolism and growth [22,23]. A previous study found that hens administered food ad libitum have elevated liver IGF1 mRNA and protein levels compared to those of restricted-diet hens [24]; egg production was lower for ad libitum-diet hens [6]. IGF1 signaling plays an important role in follicular growth and maturation. IGF1 can increase progesterone production and expression of StAR, CYP11A1, and 3βHSD in chicken granulosa cells at the preovulatory follicle stage [25,26], while increasing cell proliferation in pre-hierarchal follicles [27,28]. Female mice lacking IGF1 are infertile, and follicular development is arrested at the small antral stage [29,30]. In addition, upstream regulator analysis revealed that IGF1 is an upstream regulator in granulosa cells of 6–8 mm follicles, which influences INHA, CYP11A1, StAR, and NR5A [6]. Therefore, we speculated that the high levels of IGF1 in female geese at the early stage of the first egg-laying cycle could increase the proliferation level of granulosa cells, promote the development of pre-hierarchal follicles, and select two or more follicles for preovulatory follicles.

## 5. Conclusions

**Figure 7:**
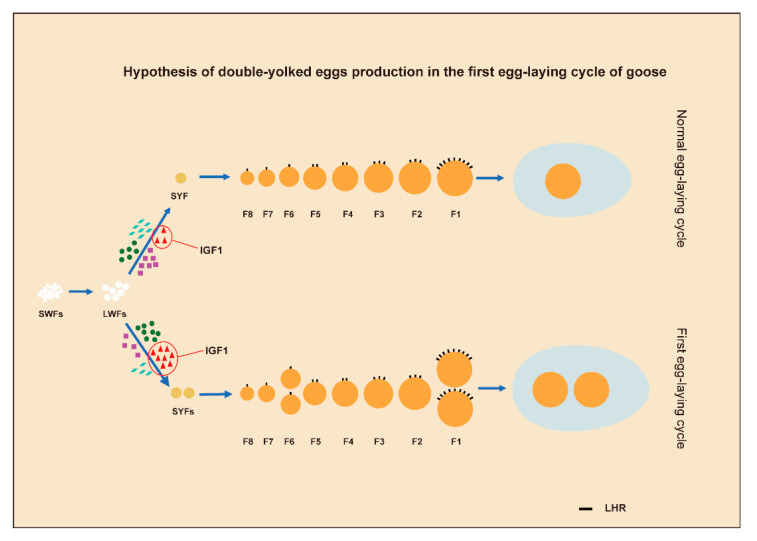
A schematic model of proposed roles of IGF1 and LH signals in producing double-yolked eggs.

In conclusion, we propose that, at the early stage of laying the first clutch egg, as a result of supplus nutritional status, geese have a high IGF1 concentration which can promote the development of pre-hierarchal follicles. Thus, two or more follicles can be selected to be preovulatory follicles at the same time. The follicles selected at the same time have similar diameters and are subjected to the same LH signal intensity before ovulation. Finally, they enter the oviduct and uterus to form the DY eggs (Figure 7).

## Figures and Tables

**Table 1:** Primer sequences used in this study.

Primers Name	GeneBank Accession	Sequences (5′-3′)	PCR Products (bp)
LHR	NM_204936.1	F:CGGATACACAACGATGCCCT	74
		R:GACTCCAGTGCCGTTGAAGA	
StAR	KF958133.1	F:GGAGCAGATGGGAGACTGGA	91
		R:CGCCTTCTCGTGGGTGAT	
IGF1R	XM_013181823.1	F:CATGTGGTTCGGTTGCTTGG	198
		R:GAGGTATGCCATCCCGTCAG	
IGFBP1	XM_013197496.1	F:GCTGTGTGCTGGTGTGTCTA	83
		R:TGTTGGCATTCAGGGTCTCC	
IGFBP3	XM_013197497	F:ATGTCTTGAGTCCCAGGGGT	89
		R:TCGACCTTTGGATGGACGAC	
γ-DH	NM_204305.1	F:GCCATCACAGCCACACAGA	120
		R:TTTCCCCACAGCCTTAGCA	

## Data Availability

Not applicable.
